# Response to the Formal Letter of Z. Chrzanowska-Lightowlers and R. N. Lightowlers Regarding Our Article “Ribosome Rescue and Translation Termination at Non-Standard Stop Codons by ICT1 in Mammalian Mitochondria”

**DOI:** 10.1371/journal.pgen.1005218

**Published:** 2015-06-18

**Authors:** Nono Takeuchi, Knud H. Nierhaus

**Affiliations:** 1 Department of Medical Genome Sciences, Graduate School of Frontier Sciences, University of Tokyo, Kashiwanoha, Kashiwa-shi, Chiba, Japan; 2 Institut für Medizinische Physik und Biophysik, Charité - Universitätsmedizin Berlin, Berlin, Germany; Max Planck Institute for Biology of Ageing, GERMANY

## Introduction

Most deviations from the universal genetic code exist in the mitochondrial translation system. In human mitochondria, two arginine codons, AGA and AGG, have no cognate tRNAs; mtDNA-encoded cytochrome c oxidase subunit I (*MTCO1*) and NADH dehydrogenase 6 (*MTND6*) carry AGA and AGG codons at the end of their mRNAs, respectively. We previously demonstrated in vitro the possible engagement of ICT1 in the translation termination at non-standard stop codons of *MTCOI* and *MTND6* mRNAs. On the other hand, Temperley and colleagues proposed in 2010 that human mitoribosomes invoke a -1 frameshift at the terminal AGA/AGG codons placing standard UAG stop codon in the ribosomal A-site. As consequence, only a single release factor, mtRF1a/RF1Lmt, would be used in mitochondria. Here we revisit the frameshift model and explain the view that ICT1 is presently a plausible candidate for the termination factor for non-standard stop codon in human mitochondria.

### Response

We previously demonstrated in vitro that the mitochondrial factor ICT1 is possibly involved in the translation termination at non-standard termination codons AGA and AGG of two mitochondrial ORFs, *MTCOI* and *MTND6*, respectively [1]. These findings challenged the in vivo data by Temperley et al., who proposed a -1 frameshift at the terminal AGA and AGG codons in *MTCOI* and *MTND6* [2]. As a consequence, both ORFs terminate in the standard UAG codon, thus using only a single release factor, mtRF1a/RF1Lmt, in mitochondria [3,4]. This model would imply that ICT1 is not necessary and is not involved in the termination of *MTCOI* and *MTND6*, since mtRF1a/RF1Lmt acts more predominantly than ICT1 at the standard UAG stop codon [1]. Here we would like to revisit the frameshift model and the mechanism of translation termination at non-standard stop codon in human mitochondria.

### Bacterial RelE is thought to show sequence preference for standard termination codons UAG and UAA and negligible recognition of AGA and AGG

An elegant technique was applied, demonstrating -1 frameshifting in human mitochondria: the bacterial RelE, an endoribonuclease that specifically cleaves mRNA in the ribosomal A-site, was targeted to mitochondria (mtRelE) [2]. In vitro studies revealed that RelE induced cleavage most commonly after the second nucleotide of the A-site codon [5]. The mtRelE cleavage occurred in *MTCO1* mRNAs uniquely between nucleotides A and G (U A|GA), suggesting a -1 frameshift with the UAG codon at the A-site.

There are two problems with this interpretation. (i) An extensive in vivo study analyzed 196 cleavage sites and observed a very modest sequence and site specificity: slightly more cuts were observed after a codon than between the second and third nucleotide of an A-site codon; 64% of the cleaved codons ended with a G or C, in 40% of the cases the cleavage occurred before or after a G [6]. In vitro study demonstrated that the bacterial RelE cleaves mRNA with high codon specificity with preference for the stop codon UAG, while the recognition of AGA codon is below the detection limit [5]. Structural study revealed that binding of RelE to ribosomal A-site reorganizes the mRNA of the A-site. Stacking of A-site codon bases with conserved residues in RelE and 16S rRNA explains the sequence specificity of the reaction, preference for pyrimidines in the first position of the codon, and purines in positions two and three [5,7]. Altogether, these data suggest that a RelE cleavage pattern cannot be taken as a proof for an A-site location of a codon as Temperley et al. do [2]. (ii) A -1 frameshift will only shift a UAG stop codon into the A-site, if the terminal AGA/AGG codon is preceded by a U nucleotide. This is not the case in most of the vertebrates, i.e., a -1 frameshift in the mitochondrial mRNAs is not universal. Furthermore, in *Oryctolagus cuniculus* (rabbit) the two terminal codons for CYTB and ND6 are UGA AGG and GCU AGG [8]; should in the first case ICT1 make the termination job, and in the second a -1 frameshift in cooperation with RF1Lmt/mtRF1a?

We think it would be important first to examine the codon specificity of RelE on 55S mitoribosome, before it is established that human mitoribosomes invoke frameshifting.

### The elements that promote -1 frameshift are ambiguously identified in COI and ND6 mRNAs

Particular elements that promote classical frameshifting have been well characterized in other systems [9–11]. These include (i) a ribosome that has paused or stalled as a consequence of either a rare or slowly decoded codon, (ii) an upstream heptapeptide “slippery sequence” followed by an inhibitory secondary structure downstream, and (iii) only an inhibitory secondary structure downstream from the frameshift site. In this regard, Temperley and colleagues invoked the following features that should provoke a -1 frameshifting in *MTCOI/MTND6* mRNA: (a) mitoribosomes stalling at AGA/AGG codons for which there are no cognate mt-tRNAs, potentially in concert with (b) diminished upstream codon/anticodon interactions due to a “non-conventional E-site,” and (c) a secondary structure immediately downstream (see supporting online material for [2]).

Limited information for the structure of mitoribosome was available when the frameshifting model was proposed. At that time, no tRNA had been found at the putative E-site of the 55S mitoribosome, and it was claimed that a conventional E-site does not exist in mitoribosomes, reducing both the number and hence overall strength of codon/anticodon interactions [12,13]. Recent structural analyses of mitoribosome, however, revealed that the binding pocket for the CCA-3’ end of the E-site tRNA observed in bacteria is well conserved in the 39S mitoribosomal large subunit and in a similar conformation compared to the occupied bacterial E-site [14]. An endogenous tRNA that co-purified with the mitoribosome can be seen in the E-site, along with the L1 stalk that interacts with it [15]. The 55S mitoribosome contains a conventional E-site, whereas elements corresponding to the slippery sequence are still obscure.

Both *MTCOI* and *MTND6* mRNA seem to contain a secondary structure immediately downstream of AGA/AGG codons. The terminal AGA of *MTCOI* mRNA is followed by the antisense of tRNAser (UCN), and the AGG of *MTND6* mRNA is followed by UTRs that extend into the antisense of *MTND5* [16]. Folding algorithms predict the antisense tRNAser (UCN) to generate a stable cloverleaf similar to a tRNA, and the sequence immediately downstream of *MTND6* to form a stem loop (RNAfold Web Server, http://rna.tbi.univie.ac.at/cgi-bin/RNAfold.cgi). It is of note here that these sequences immediately downstream of AGA/AGG codons cannot fold into structure when the ribosome is paused at AGA/AGG codon. Accordingly, it is still unclear whether a secondary structure immediately downstream of AGA/AGG codons functions to promote -1 frameshifting.

At present, ICT1 is the only factor that has been shown to exhibit a peptide release activity at non-standard stop codons AGA and AGG. The structural model of ICT1 has also supported this peptide release activity of ICT1 at non-standard stop codons [8]. The integrated ICT1 is located at the base of the central protuberance >70 Å away from the A-site [14,15,17] and, therefore, cannot act as a termination factor at the A-site. It is still an exciting riddle, whether or not the integrated ICT1 is released from the ribosome under distinct condition to fulfill its termination function. A similarly exciting question is the role of the other members of the mitochondrial RF family, namely mtRF1 and C12orf65. It is still possible that mtRF1 and/or C12orf65 are involved in the termination of non-standard stop codons in mammalian mitochondria.
